# The nature of the Syntaxin4 C-terminus affects Munc18c-supported SNARE assembly

**DOI:** 10.1371/journal.pone.0183366

**Published:** 2017-08-25

**Authors:** Asma Rehman, Shu-Hong Hu, Zakir Tnimov, Andrew E. Whitten, Gordon J. King, Russell J. Jarrott, Suzanne J. Norwood, Kirill Alexandrov, Brett M. Collins, Michelle P. Christie, Jennifer L. Martin

**Affiliations:** 1 Division of Chemistry and Structural Biology, Institute for Molecular Bioscience, The University of Queensland, St Lucia, Brisbane, QLD, Australia; 2 Division of Cell Biology and Molecular Medicine, Institute for Molecular Bioscience, The University of Queensland, St Lucia, Brisbane, QLD, Australia; University of Cincinnati College of Medicine, UNITED STATES

## Abstract

Vesicular transport of cellular cargo requires targeted membrane fusion and formation of a SNARE protein complex that draws the two apposing fusing membranes together. Insulin-regulated delivery and fusion of glucose transporter-4 storage vesicles at the cell surface is dependent on two key proteins: the SNARE integral membrane protein Syntaxin4 (Sx4) and the soluble regulatory protein Munc18c. Many reported *in vitro* studies of Munc18c:Sx4 interactions and of SNARE complex formation have used soluble Sx4 constructs lacking the native transmembrane domain. As a consequence, the importance of the Sx4 C-terminal anchor remains poorly understood. Here we show that soluble C-terminally truncated Sx4 dissociates more rapidly from Munc18c than Sx4 where the C-terminal transmembrane domain is replaced with a T4-lysozyme fusion. We also show that Munc18c appears to inhibit SNARE complex formation when soluble C-terminally truncated Sx4 is used but does not inhibit SNARE complex formation when Sx4 is C-terminally anchored (by a C-terminal His-tag bound to resin, by a C-terminal T4L fusion or by the native C-terminal transmembrane domain in detergent micelles). We conclude that the C-terminus of Sx4 is critical for its interaction with Munc18c, and that the reported inhibitory role of Munc18c may be an artifact of experimental design. These results support the notion that a primary role of Munc18c is to support SNARE complex formation and membrane fusion.

## Introduction

Vesicular trafficking in eukaryotic cells depends on targeted fusion reactions between vesicles and their specific target membranes. Two universally required components of the intracellular membrane fusion machinery are soluble N–ethylmaleimide-sensitive factor attachment protein receptor (SNARE) proteins and Sec1/Munc18 (SM) proteins. Target membrane (*t*-) SNAREs, anchored to one membrane, form a molecular zipper with cognate vesicle (*v*-) SNAREs on a vesicle membrane generating an α-helical bundle that pulls the two membranes together and drives fusion [1–3].

Vesicular transport is dependent on SNARE complex assembly, which is regulated by SM proteins [4, 5]. Thus, delivery of the glucose transporter-4 (GLUT4) to the cell membrane in response to insulin signaling requires the SM protein Munc18c as well as two *t*- SNAREs (Syntaxin4 (Sx4), SNAP23) [6, 7] and the *v*-SNARE (VAMP2/syntaptobrevin) [8]. Knockouts and overexpression of SM proteins have shown both positive and negative effects on SNARE complex assembly and vesicle fusion [9, 10], and there is little agreement on the precise role of SM proteins.

Three isoforms of SM proteins have been identified for exocytosis events in mammals: Munc18a (also known as Munc18-1), Munc18b (Munc18-2) and Munc18c (Munc18-3) [7, 11]. Each Munc18 protein interacts with a cognate Syntaxin (Sx) protein composed of an N-peptide (a 10–30 residue region at the N-terminus) followed by an α-helical bundle (the H_abc_ domain), a SNARE motif, and a C-terminal transmembrane region. The best-studied Munc18:Sx interaction is that between Munc18a and Syntaxin1a (Sx1a); both are required for synaptic vesicle-mediated neurotransmitter release [2, 12, 13]. Munc18a interacts with Sx1a with nanomolar affinity [14–18] and two alternate binding modes have been described. The first involves Munc18a interacting with a “closed” 4-helix bundle conformation of Sx1a (where the SNARE helix is sequestered by the H_abc_ domain). This Munc18a:Sx1a closed binding mode is consistent with a negative regulatory role for Munc18a, because the SNARE helix is unable to interact with partner SNAREs to drive vesicle fusion [15, 16, 18–20]. A second Munc18a:Sx1a binding mode occurs when Munc18a associates with the SNARE ternary complex in which Sx1a adopts an “open” conformation [17, 21–26]. While the neuronal Munc18a:Sx1a complex is reported to form closed “fusion-incompetent” and open “fusion-competent” conformations, the closely related Munc18c:Sx4 system appears to use only the open Sx4 binding mode [17, 27, 28], though this remains controversial. Indeed, the closed binding mode interaction of Sx1a with Munc18a has been suggested to be a specialization of the neuronal exocytotic pathways [29].

**Fig 1:**
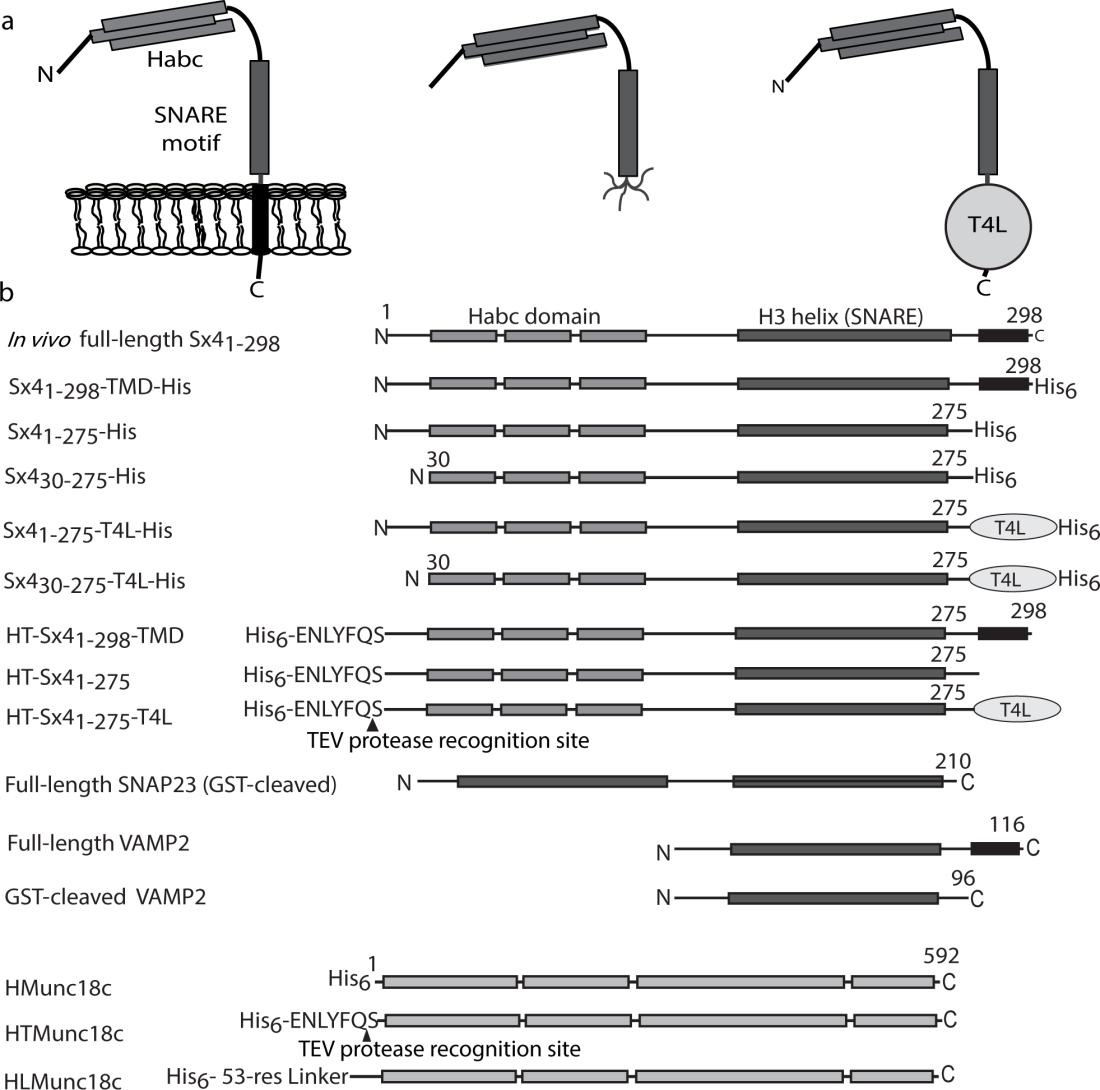
Schematic of protein constructs used in this study. **a.** In vivo full-length Sx4_1-298_ consists of an N-terminal peptide, an α-helical bundle (the H_abc_ domain), a SNARE motif and a C-terminal transmembrane domain. Without the transmembrane domain Sx4 is no longer associated with a membrane and the Sx4 SNARE helix is also likely to be flexible when it is not anchored. We investigated the effect of anchoring the Sx4 C-terminus by using full-length Sx4 including the transmembrane domain, soluble Sx4 lacking the transmembrane domain and soluble Sx4 with a C-terminal T4 lysozyme (T4L) fusion. **b.** Schematic representation of all protein constructs used in this study, illustrating the position of engineered fusion tags and protease cleavage sites. For Sx4 constructs, His indicates a C-terminal hexahistidine (His_6_) tag, TMD indicates a C-terminal transmembrane domain, HT indicates an N-terminal His_6_ tag with a TEV protease recognition site. For Munc18c, H indicates an N-terminal His_6_ tag, HT indicates an N-terminal His_6_ tag with a TEV protease recognition site, HL indicates an N-terminal His_6_ tag with a long linker. SNAP23 and VAMP2 proteins used in this study are the constructs labeled GST-cleaved in this figure.

In the cell, Sxs are anchored by a C-terminal transmembrane helix to the plasma membrane, providing an anchor for the adjoining SNARE helix (**Fig 1A**). Furthermore, a crystal structure of the neuronal SNARE complex shows that the C-terminal transmembrane domains of Sx1a and VAMP2 form continuous α-helices with their SNARE motifs [30]. This finding suggests that the transmembrane domain is not only an anchor but may also play an integral part in the protein structure. However, *in vitro* experiments designed to study Munc18:Sx interactions, both in the neuronal and GLUT4 systems, have generally used truncated Sxs, including soluble Sx lacking its C-terminal membrane anchor [15, 18], soluble truncated Sx immobilized at its N-terminus (with the SNARE helix untethered) [31] or soluble Sx immobilized at its C-terminus [17, 25, 28]. Other reports have used full-length Sxs incorporated into proteoliposomes through native C-terminal membrane anchors [23, 24, 27, 32–36]. Based on the conclusions derived from these experiments we postulated that the nature of the Sx4 C-terminus affects the observed *in vitro* activity of Munc18c [37]. Here, we test the hypothesis that experimental design–specifically focusing on the Sx4 C-terminus—influences the observed effect of Munc18c on SNARE assembly. We show that when the Sx4 C-terminus is anchored, SNARE assembly occurs in the presence of Munc18c yet when the Sx4 C-terminus is not anchored SNARE assembly is inhibited in the presence of Munc18c.

## Results

To investigate whether anchoring of the Sx4 C-terminus has an effect on SNARE complex assembly in the presence of Munc18c, we designed several Sx4 constructs including: the soluble cytoplasmic domain (Sx4_1-275_); a Sx4 construct containing the soluble cytoplasmic domain with a C-terminal fusion protein T4-lysozyme (Sx4_1-275_-T4L); and the full-length protein complete with C-terminal transmembrane domain helix (Sx4_1-298_-TMD). We hypothesized that C-terminal anchoring of the Sx4 constructs may mimic the *in vivo* situation where Sx4 is inserted into the plasma membrane by its C-terminal transmembrane helix. The Sx4_1-275_-T4L protein fusion was included as a way of reducing the Sx4 C-terminal flexibility that is likely to occur when the transmembrane domain is removed, and potentially structure the otherwise disordered C-terminal region in Sx4_1-275_ [17]. A similar strategy has been used successfully to decrease the flexibility of loops and termini in GPCR research [38]. A range of Sx4 constructs were engineered with either a C-terminal His_6_-tag for purification and immobilization on metal affinity resin, or a TEV-cleavable N-terminal His_6_-tag for the production of de-tagged Sx4 proteins. The Sx4, Munc18c, SNAP23 and VAMP2 constructs used in this work are shown schematically in **Fig 1B.**

### C-terminally anchored Sx4 with a T4L fusion forms a complex with Munc18c

**Fig 2:**
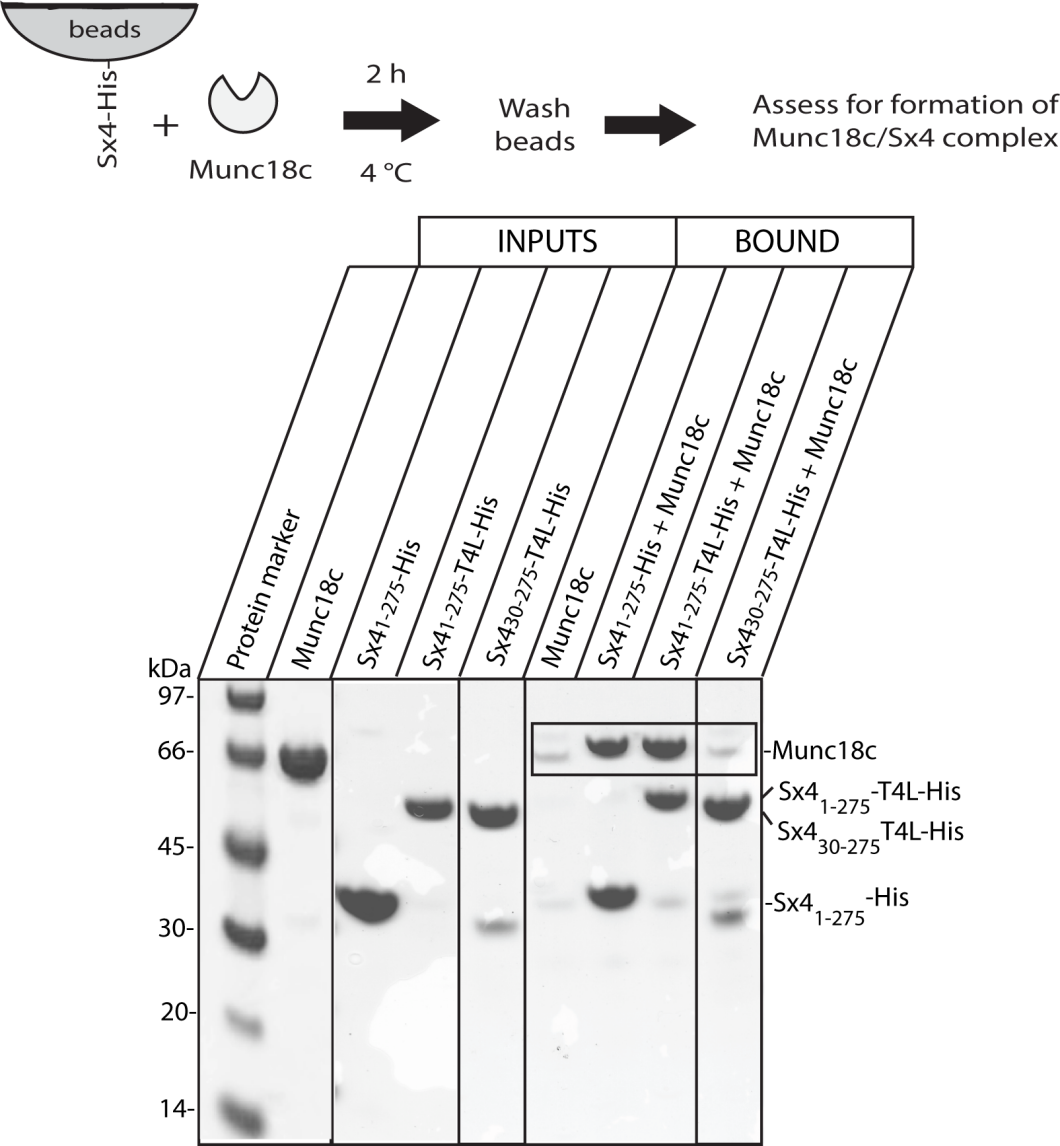
Munc18c binds to Sx4 proteins immobilized by their C-terminus onto beads. Coomassie Blue stained SDS-PAGE gel showing that (after a 2 hr incubation at 4°C) de-tagged Munc18c is pulled down by all Sx4 proteins immobilized by their C-terminus, except Sx4_30-275_-T4L which lacks the N-peptide sequence known to be important for Munc18c binding. There was negligible non-specific binding of de-tagged Munc18c to the metal affinity resin (lane labeled Bound, Munc18c). The expected position of Munc18c on the gel is boxed. The gel displayed is representative of three replicate experiments. Solid vertical lines on the gel image indicate the removal of intervening lanes.

We undertook pulldown experiments to investigate if the Sx4_1-275_-T4L construct behaved similarly to Sx4_1-275_. Sx4 constructs were immobilized via their C-terminal His_6_-tag onto metal affinity resin, so that all Sx4 constructs were similarly bound by their C-terminus. The results of these experiments clearly show that Munc18c binds to both C-terminally immobilized Sx4 proteins Sx4_1-275_-His and Sx4_1-275_-T4L-His (**Fig 2)**. Importantly, only a very small amount of Munc18c was pulled down in the negative control (labeled Munc18c), confirming that the binding of Munc18c was specific to Sx4. Moreover, no Munc18c above the negative control levels was pulled down by Sx4_30-275_-T4L-His, consistent with previous observations that binding of Munc18c to Sx4 requires the Sx4 N-terminal residues (the Syntaxin N-peptide) [17, 28].

The importance of the Sx4 N-peptide for binding to Munc18c was confirmed once again using ITC, where binding was detected for HMunc18c (Munc18c with an N-terminal His_6_-tag) and Sx4_1-275_-T4L-His, but not for HMunc18c and Sx4_30-275_-T4L-His (**S1 Table**). The ITC determined binding affinity, *K*_d_, of 100 nM for the HMunc18c:Sx4_1-275_-T4L-His interaction (**S1 Table, S1 Fig**) is similar to that reported previously for the interaction between Munc18c and Sx4_1-275_-His (*K*_d_ 95–104 nM) [17, 39]. These results indicate that the T4L fusion does not affect the binding affinity of Sx4 for Munc18c.

### C-terminal anchoring of Sx4 slows dissociation from Munc18c

To assess more quantitatively the effect of anchoring the C-terminus of Sx4, we assayed Munc18c interactions with Sx4 constructs using bio-layer interferometry. When Sx4 constructs were immobilized via their C-terminal His_6_-tag onto Ni-NTA biosensors, both Sx4 variants—Sx4_1-275_-His and Sx4_1-275_-T4L-His—had similar association rate constants (*k*_on_, M^-1^s^-1^), dissociation rate constants (off-rates, *k*_off_, s^-1^) and binding affinities (**Table 1, S2 Fig**) for detagged Munc18c. In the reverse experiment, HLMunc18c was immobilized. In this case, Sx4_1-275_-T4L (*K*_d_ = 31 nM) bound to HLMunc18c with a higher affinity than Sx4_1-275_ (*K*_d_ = 72 nM). Further analysis showed that the two pairs of proteins had similar association rate constants, but that the dissociation rate constant for HLMunc18c:Sx4_1-275_ was ~2.5 times faster than for HLMunc18c:Sx4_1-275_-T4L demonstrating a weaker association for the former.

**Table 1:** Summary of kinetic data for the Munc18c-Syntaxin4 interaction. Values shown for the bio-layer interferometry experiments are mean ± s.d. where each experiment was repeated at least three times. Values and standard errors are shown for the fluorescence anisotropy experiments and these were obtained from a global fit to multiple kinetic data sets with differing concentrations of Munc18c. Bold text indicates dissociation rate constants (k_off_) for Munc18c-Sx4_1-275_ where Sx4_1-275_ is free in solution.

Sx4 construct	Experimental design		*k*_on_, M^-1^s^-1^	*k*_off_, s^-1^	*K*_d_, nM
	Sx4	Munc18c			
**Bio-layer interferometry**					
Sx4_1-275_-His	Immobilized	In solution	(5.3 ± 2.6) × 10^5^	(8.6 ± 1.1) ×10^−3^	21 ± 13
Sx4_1-275_-T4L-His	Immobilized	In solution	(4.3 ± 1.8) × 10^5^	(7.8 ± 2.5) ×10^−3^	20 ± 7
Sx4_1-275_	In solution	Immobilized	(3.0 ± 0.7) ×10^5^	**(22 ± 8) ×10**^**−3**^	72 ± 16
Sx4_1-275_-T4L	In solution	Immobilized	(2.9 ± 0.4) × 10^5^	(9.0 ± 1.2) × 10^−3^	31 ± 6
**Fluorescence Anisotropy**					
Sx4_1-275_-His	In solution	In solution	(1.30 ± 0.04) ×10^3^	**(65.3 ± 0.4) ×10**^**−5**^	502 ± 16
Sx4_1-275_-T4L-His	In solution	In solution	(5.02 ± 0.13) × 10^2^	(7.5 ± 0.4) × 10^−5^	148 ± 9

**Fig 3:**
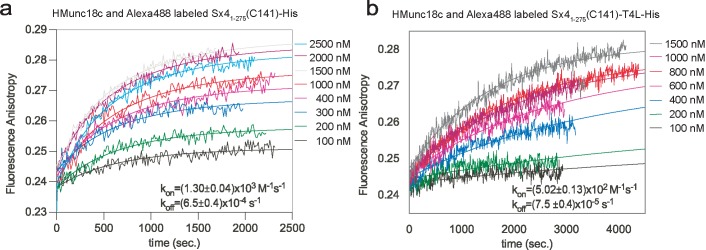
Kinetics of Munc18c interaction with Sx4 in solution. **a.** Time course of complex formation of Alexa488-labeled Sx4_1-275_-His (50 nM) with HMunc18c at various concentrations: 100 nM (black curve), 200 nM (green curve), 300 nM (blue curve), 400 nM (pink curve), 1000 nM (red curve), 1500 nM (grey curve), 2000 nM (magenta curve) and 2500 nM (cyan curve). Solid lines represent global fits to titration data in the Dynafit 4.0 program. Resulting kinetic constants are shown in the graph inset **b**. As for **a**. except that formation of HMunc18c:Sx4_1-275_-T4L-His was monitored. Concentrations of HMunc18c used were: 100 nM (black curve), 200 nM (green curve), 400 nM (blue curve), 600 nm (pink curve), 800 nM (red curve), 1000 nM (magenta curve), 1500 nM (gray curve).

We then investigated these same pairs of interactions by fluorescence anisotropy where neither protein is immobilized. The Sx4 proteins (Sx4_1-275_-His, Sx4_1-275_-T4L-His) were labeled with the Alexa Fluor® 488 maleimide probe (see Materials and Methods) and kinetic titrations of the labeled Sx4 proteins (each at 50 nM) were measured with increasing concentrations of HMunc18c. Addition of HMunc18c to the Alexa488-labeled Sx4_1-275_-His or Sx4_1-275_-T4L-His resulted in an increase in fluorescence anisotropy indicating formation of Munc18c complexes with both Sx4 variants (**Fig 3A and 3B**). The T4L fusion did not greatly affect the association rate constant. In contrast, the dissociation rate constant (*k*_off_, s^-1^) of HMunc18c:Sx4_1-275_-His was almost an order of magnitude faster than that of HMunc18c:Sx4_1-275_-T4L-His (**Table 1**). These results are consistent with the outcomes from the bio-layer interferometry experiments and support the notion that the nature of the Sx4 C-terminus affects dissociation kinetics with Munc18c.

In combination, these results show that anchoring the C-terminus of Sx4 (either by using a C-terminal His-tag anchored to affinity resin or by using a C-terminal T4L fusion) slows dissociation of Sx4 from Munc18c, although the mechanism for this is unclear.

### Sx4 C-terminal anchoring promotes SNARE assembly in the presence of Munc18c

**Fig 4:**
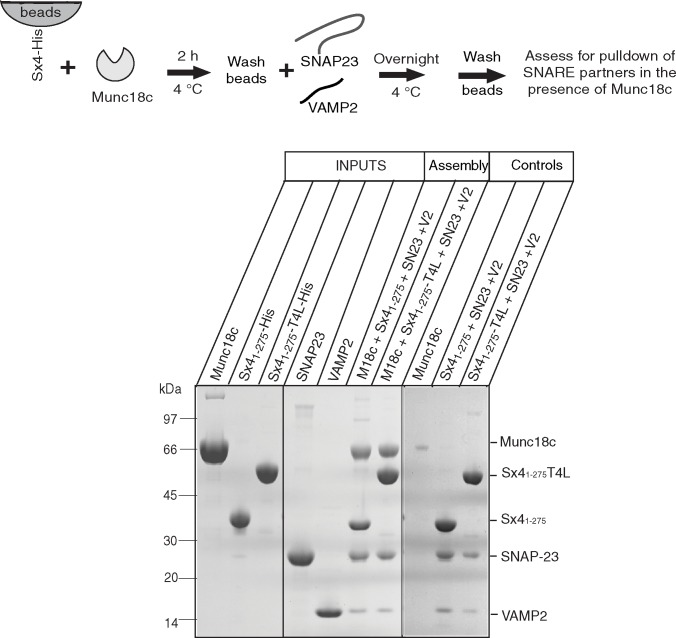
Munc18c does not block SNARE assembly when the Sx4 C-terminus is immobilized. Coomassie Blue stained SDS-PAGE analysis of the binding of SNAP23 and VAMP2 to Sx4 proteins (Sx4_1-275_-His or Sx4_1-275_-T4L-His) immobilized on Co^2+^resin by their C-terminus. Immobilized Sx4 proteins were complexed with Munc18c (de-tagged) prior to overnight incubation with SNAP23 and VAMP2. C-terminally immobilized Sx4 proteins were able to pull down SNARE partners (SNAP23 and VAMP2) in the presence of Munc18c (lanes labeled Assembly) and in the absence of Munc18c (lanes labeled Controls). Lanes labeled INPUTS show the protein samples used in the experiments. The control lane labeled Munc18c is a negative control showing the lack of interaction of Munc18c with beads. The gel displayed is representative of three replicate experiments. Solid vertical lines on the gel image indicate the removal of intervening lanes or where two different gels have been placed adjacent to each other.

We next investigated the effect of Sx4 C-terminal anchoring on the impact of Munc18c-mediated SNARE complex assembly. Using a pull-down assay, we explored the ability of Sx4 variants (Sx4_1-275_-His and Sx4_1-275_-T4L-His) to bind SNARE partners. Both Sx4 constructs, when immobilized on metal affinity resin by their C-terminal His_6_-tags in a pre-formed complex with Munc18c, were able to pull down SNAP23 and VAMP2 to form SNARE complexes after overnight incubation (**Fig 4**).

**Fig 5:**
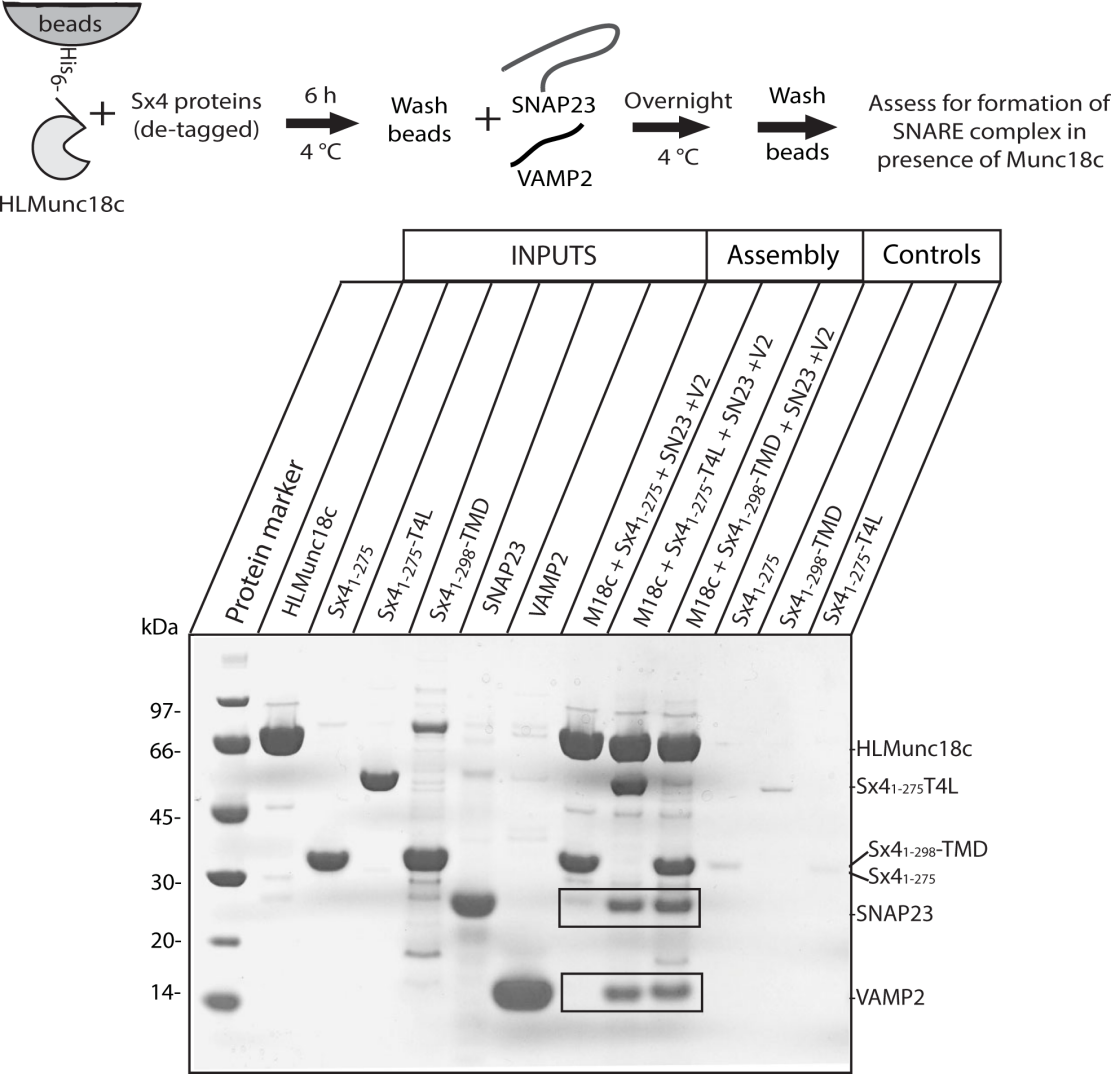
Munc18c prevents SNARE assembly when Sx4 C-terminus is not immobilized. Coomassie Blue stained SDS-PAGE analysis of the binding of SNAP23 and VAMP2 to Sx4s (de-tagged Sx4_1-275_, Sx4_1-275_-T4L or Sx4_1-298_-TMD) bound to HL-Munc18c. HL-Munc18c was immobilized on Co^2+^resin and incubated for 6 h with Sx4_1-275_, Sx4_1-275_-T4L or Sx4_1-298_-TMD and then allowed to interact with SNAP23 and VAMP2 overnight. The gel shows that SNAP23 and VAMP2 were pulled down by Munc18c:Sx4_1-275_-T4L and Munc18c:Sx4_1-298_-TMD but not by Munc18c:Sx4_1-275_. The expected positions of pulled down SNAP23 and VAMP2 proteins are boxed. The gel displayed is representative of four replicate experiments. Lanes labeled INPUTS show the protein samples used in the experiments. Lanes labeled Controls, show that Sx4_1-275_, Sx4_1-275_-T4L or Sx4_1-298_-TMD do not bind to the Co^2+^resin in the absence of bound HL-Munc18c.

The importance of Sx4 C-terminal anchoring on SNARE complex assembly was then examined by using a pre-formed binary complex between immobilized HL-Munc18c and de-tagged Sx4 constructs in SNARE pull-down experiments (**Fig 5**). HL-Munc18c was first immobilized onto beads, then Sx4 proteins were pulled down. For this critical experiment, we used detagged Sx4_1-275_, Sx4_1-275_-T4L and the full-length Sx4_1-298-_TMD (in detergent micelles). After extensive washing to remove unbound Sx4 proteins, the beads were incubated with SNARE partner proteins (SNAP23 and VAMP2). We found that when bound to HL-Munc18c, both Sx4_1-275_-T4L and Sx4_1-298_-TMD were able to assemble ternary complex by pulling down SNAP23 and VAMP2, whereas Sx4_1-275_ did not (**Fig 5**). The experiments were repeated four times, including the use of different batches of each purified protein. In each experiment, the results were the same. This result shows that SNAP23 and VAMP2 are pulled down only when Munc18c is in complex with a Sx4 construct that has a C-terminal anchor. Pulldown experiments using T4L-His immobilized on beads showed negligible binding of SNAP23 or VAMP2 (**S3 Fig**) indicating that the capture of these two proteins (in the experiment presented in **Fig 5**) is due to interaction with Sx4 and not with T4L.

### Munc18c only inhibits SNARE assembly when Sx4 C-terminus is not anchored

To assess the role of Munc18c in SNARE complex formation when each cognate SNARE and SM protein is free in solution, we made use of a fluorescence anisotropy assay that has been used previously to assess neuronal SNARE assembly [15, 18]. In the previously reported work, SNARE complex formation using the soluble cytoplasmic form of neuronal Sx1a (Sx1a_1-262_) was inhibited in the presence of Munc18a. To gain insight into the importance of the Sx4 C-terminus, our experiments were designed to evaluate whether the same result occurs for Sx4_1-275_ (analogous to the cytoplasmic Sx1a_1-262_ construct) and whether this result changes when the C-terminus is fused to T4L (Sx4_1-275_ -T4L).

**Fig 6:**
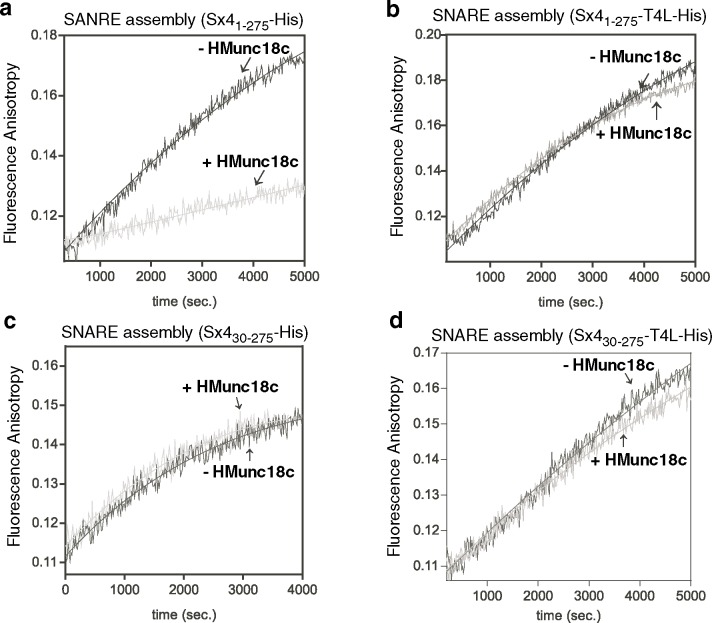
Munc18c reduces the rate of SNARE complex formation in solution when the Sx4 C-terminus is not anchored. Panels **a**. Sx4_1-275_-His, **b.** Sx4_1-275_-T4L-His, **c**. Sx4_30-275_-His and **d.** Sx4_30-275_-T4L-His show typical fluorescence anisotropy traces upon mixing SNARE complex components: 700 nM SNAP23, 100 nM Alexa488-labeled VAMP2 and 700 nM Sx4-variant alone (dark gray) or in the presence of 500 nM Munc18c (light gray).

SNARE complex formation was monitored by the change in fluorescence anisotropy of Alexa488 labeled VAMP2 in the presence or absence of Munc18c. In the absence of Munc18c, SNARE ternary complex was formed in the presence of both Sx4_1-275_-His and Sx4_1-275_ -T4L-His at almost the same rates (**Table 2).** In contrast, the rate of SNARE complex formation for Sx4_1-275_-His in the presence of HMunc18c is 7-fold lower than for Sx4_1-275_-His alone (**Table 2, Fig 6A**). These findings for Munc18c are in agreement with the results of previous studies on neuronal counterparts [15, 16, 18] that showed Munc18a inhibited assembly of Sx1a_1-262_ SNARE complexes in solution.

**Table 2:** Observed rate constants (k_obs_, s^-1^) of SNARE complex formation measured by fluorescence anisotropy (VAMP2 labeled) for Sx4 constructs in the presence and absence of Munc18c. Values are shown as mean k_obs_ ± s.d. from three independent experiments. Bold text indicates the observed rate constant (k_obs_) for Sx4_1-275_ SNARE assembly in the absence and presence of Munc18c showing that SNARE assembly is 7-fold slower in the presence of Munc18c for this construct only. Shading indicates all four experiments for which Munc18c was included, showing that Munc18c did not inhibit SNARE assembly when the Sx4 C-terminus had a T4L fusion, or when the Sx4 N-peptide was removed.

Protein interaction partners	*k*_obs_, s^-1^
**Sx4**_**1-275**_-His **SNARE assembly**	**(4.9 ± 0.9) × 10**^**−4**^
Sx4_1-275_-T4L-His SNARE assembly	(5.0 ± 1.0) × 10^−4^
Sx4_30-275_-His SNARE assembly	(4.9 ± 1.4) × 10^−4^
Sx4_30-275_T4L-His SNARE assembly	(4.8 ± 1.7) × 10^−4^
**Sx4**_**1-275**_-His **SNARE assembly + HMunc18c**	**(0.7 ± 0.4) × 10**^**−4**^
Sx4_1-275_-T4L-His SNARE assembly + HMunc18c	(5.1 ± 1.1) × 10^−4^
Sx4_30-275_-His SNARE assembly + HMunc18c	(4.8 ± 0.6) × 10^−4^
Sx4_30-275_T4L-His SNARE assembly + HMunc18c	(5.0 ± 0.8) × 10^−4^

When we conducted the same experiment using Sx4_1-275_-T4L-His the rate of SNARE complex formation is unchanged by the addition of HMunc18c (**Table 2, Fig 6B**). This outcome is in line with the results reported above from pull-down assays where Sx4_1-275_-T4L or Sx4_1-298_-TMD bound to immobilized HLMunc18c were able to pull down SNARE partners, whereas Sx4_1-275_ bound to Munc18c failed to form SNARE complexes unless the Sx4_1-275_ was C-terminally immobilized on beads.

### Removing the Sx4 N-peptide releases the effect of Munc18c on SNARE assembly

To assess whether the N-terminal peptide of Sx4 plays a role in SNARE assembly, we performed fluorescence anisotropy experiments analogous to those reported previously for Munc18a:Sx1a [18]. We studied complex formation for Sx4_30-275_ in the presence or absence of Munc18c (**Table 2, Fig 6C**). We also analyzed SNARE complex formation using Sx4_30-275_-T4L (having a C-terminal fusion) in the presence and absence of Munc18c. The rate of SNARE assembly in the absence of Munc18c is similar for Sx4_30-275_ and Sx4_1-275_ (**Table 2**) indicating that the presence of the N-peptide does not affect the interaction with SNARE partners. However there is a marked difference in the rate of SNARE assembly for Sx4_30-275_ and Sx4_1-275_ (i.e. without and with N-peptide) in the presence of Munc18c. For Sx4_1-275_, SNARE assembly is reduced by 7-fold in the presence of Munc18c. For Sx4_30-275_ there is no significant rate reduction of SNARE assembly in the presence of Munc18c (**Table 2, Fig 6C**). When the C-terminal Sx4_30-275_-T4L fusion protein is used, there is again no difference observed in the rate of SNARE assembly in the presence of Munc18c (**Table 2**, **Fig 6D**). Indeed, for Sx4s lacking N-terminal residues, SNARE assembly in solution proceeds at very similar rates whether or not the Sx4 C-terminus is fused to T4L and whether or not Munc18c is present. We note that Sx4_30-275_ binds very weakly, if at all, to Munc18c [17] (**Fig 3, S1 Table)**, so that SNARE assembly for this construct probably does not involve Munc18c. Thus the “release” of Munc18c inhibition observed when the N-peptide of Sx4 is removed, may simply be a consequence of Munc18c not participating in SNARE assembly.

## Discussion

Previously reported *in vitro* evidence for the role of Munc18c has been used to support both positive [27, 28] and negative [32, 40] regulatory roles on SNARE assembly. This situation is mirrored for the neuronal counterpart Munc18a [15, 16, 18, 19, 21–27, 33, 34, 41]. We questioned whether these contradictory conclusions for the role of Munc18c - derived from *in vitro* experiments—could be a consequence of experimental design.

Removing the Sx4 C-terminus would have two immediately apparent effects: first, Sx4 would not be associated with a membrane; and, second, the Sx4 SNARE region would not be anchored at its C-terminus and its C-terminal residues would be less ordered/more flexible. Indeed, a crystal structure of the C-terminally truncated Sx1 in complex with Munc18a revealed that the C-terminal region of Sx1 was disordered [42]. Similarly, the SNARE region of a soluble C-terminally truncated Sx1 construct was flexible [17, 43]. On the other hand, a crystal structure of the neuronal SNARE complex that includes the Sx1 and VAMP2 C-terminal transmembrane domains showed a continuous helical structure for the SNARE motifs and TMD domains [30]. Moreover, NMR analysis of Sx1 including the transmembrane domain in micelles showed two well-ordered helices in the SNARE domain and a well-ordered helix in the transmembrane region [44]. Together this evidence supports the conjecture that removing the transmembrane anchor of Sxs could affect the C-terminal SNARE structure. Here we investigated whether this effect might impact on the outcome of *in vitro* SNARE assembly experiments.

Using multiple lines of evidence, we found a consistent relationship. In essence, different experimental designs give rise to contradictory conclusions on the role of Munc18c in SNARE assembly. Strategies in which the Sx4 C-terminus is attached to beads in *in vitro* pull-down assays show that Munc18c does not inhibit SNARE assembly. Conversely, strategies in which Sx4 is not immobilized by its C-terminus or is free in solution show that SNARE assembly is inhibited when C-terminally truncated Sx4 is used, but not when using Sx4 that includes its TMD, or when fused to T4L. The *in vitro* experimental design using a C-terminal anchor (T4L or TMD in a detergent micelle) does not include a membrane bilayer, but nevertheless the outcomes closely resemble those reported for Sx4 reconstituted in liposomes [27].

Unlike neuronal Munc18a, Munc18c is thought to only bind Sx4 in an open conformation [17, 27]. The inclusion of a Sx4 C-terminal anchor in *in vitro* experiments may enable a conformation of Sx4 in the Munc18c:Sx4 complex that can also accommodate SNAP23 and VAMP2 binding. This finding is of critical importance in the field, and explains conflicting conclusions from studies of the role of Munc18c in promoting SNARE assembly. In its native form Sx4 is membrane-embedded. Our results thus provide compelling evidence that Munc18c does not inhibit SNARE assembly *in vivo*. We reason that the negative regulatory role ascribed to Munc18c is artifactual and is a consequence of using soluble truncated Sx4. However, further experiments will now be required to define the precise role of the Sx4 C-terminal region in modulating Munc18c/SNARE interactions.

More broadly, our findings suggest that experimental design and interpretation of *in vitro* data should perhaps be revisited for other vesicle fusion systems. We note, for example, that *in vivo* studies using Sx1 with a lipidic anchor in place of the transmembrane helix fully supported fusion, but that distancing the SNARE motif from the membrane inhibits membrane fusion [45], in support of our model. Our findings suggest that the most appropriate Sx construct to use for *in vitro* experiments with Munc18 partners is the membrane-anchored form or, failing that, Sx with a C-terminal tag attached to affinity beads, or a C-terminal fusion such as T4L.

In summary, our data show that Munc18c only has an inhibitory effect on SNARE assembly when the Sx4 C-terminus is removed. Because native Sx4 is anchored by its C-terminus to the plasma membrane, we conclude that Munc18c almost certainly does not block native Sx4 SNARE assembly *in vivo*. Other membrane fusion components may exert complex regulatory control in these exquisitely organized trafficking systems, including a wide range of positive and negative regulation of Munc18 proteins by a wide range of mechanisms. Finally, our findings call into question the common practice of removing transmembrane anchors to study protein interactions when the interacting region of the protein adjoins the transmembrane helix.

## Materials and methods

### Constructs

Plasmids encoding rat Sx4 (C141S, amino acids 1–275), with a C-terminal hexahistidine (His_6_) tag, and the glutathione S-transferase (GST) fusion proteins, mouse SNAP23 (amino acids 1–210), rat VAMP2 (amino acids 1–96) were generated as described previously [17, 28]. Mouse HMunc18c (N-terminal His_6_-tag), HTMunc18c (N-terminal His_6_-tag, TEV cleavage site, referred to as detagged Munc18c after the His tag is removed) and HL-Munc18c (N-terminal His_6_-tag, with a 53 amino acid linker) constructs were also prepared as described by Rehman *et al*. [39]. The 53 amino acid linker has the following sequence: MSPIDPMGHHHHHHGRRASVAAGILVPRGSPGLDGIYARGIQASMAAGFG. For production of T4L fusion constructs, a synthetic gene for T4L (amino acids 1–164) was purchased from GeneArt® Gene Synthesis (Life Technologies, Carlsbad, CA) and cloned into the pET20b vector using the *HindIII* and *XhoI* restriction sites. The cytoplasmic Sx4 constructs (amino acids 1–275 and 30–275) were subsequently ligated into the pET20b-T4L vector between the *NdeI* and *HindIII* sites, resulting in the constructs designated Sx4_1-275_-T4L-His and Sx4_30-275_-T4L-His. Constructs containing a TEV cleavage site were generated by ligation independent cloning (LIC) [46]. TEV cleavage was required to produce de-tagged protein for the analysis of the interaction between unbound Sx4 and Munc18c, and unbound Sx4 and SNARE proteins. To generate constructs with an N-terminal cleavable His-tag, a ligation independent cloning (LIC) strategy was used [46]. For this, each fragment i.e. Sx4_1-275_, Sx4_1-275_-T4L, Sx4_1-298_-TMD or Munc18c was PCR amplified using specific forward **(****5’-**CAGGGCGCCATG**CGCGACAGGACCCATGAGTTGAGGC-3’****)** and reverse **(****5'-**GACCCGACGCGGTA**AGTGAGCTCCAGGTTTTTATACGCAT-3'****)** primers containing LIC overhangs (in bold). The PCR product was then treated with T4-polymerase. T4 polymerase treated DNA was ligated to a LIC vector (pMCSG7 vector digested with Ssp1 restriction enzyme). The LIC vector pMCSG7 encodes an N-terminal leader sequence containing a His_6_ fusion tag and a TEV protease site (EXXYXQ**G/S**). The plasmid was isolated and the sequence confirmed (Australian Genome Research Facility, University of Queensland).

To generate HT-Sx4-TMD a synthetic gene encoding full length Sx4-TMD (mouse, amino acids 1–298) was purchased from GenScript® (Piscataway, NJ) and cloned into a LIC vector with an N-terminal His_6_ affinity tag as described above.

### Production of soluble Sx, SNAP23, VAMP2 and Munc18 constructs

Munc18c proteins were expressed in *E*. *coli* and purified as described previously [39], with the following changes. Tris was replaced with 50 mM phosphate (pH 8.0) in all purification buffers. Cell pellet from 1 L of cells (OD ~18) was lysed using 500 mg lysozyme in lysis buffer (300 mL) at room temperature. The soluble forms of Sx4 proteins and its T4L fusions were expressed in *E*. *coli* and purified using the methods described for Sx4 previously [17, 28]. To remove the His_6_-tag, proteins eluted after immobilized metal ion affinity chromatography (IMAC) with TALON™ Co^2+^ resin were mixed with TEV protease (A_280_ ratio of 1:100, protease: protein) and incubated overnight at 4°C. The following day, the protein was subjected to a reverse IMAC step and cleaved protein collected in the flow-through was further purified on a size exclusion chromatography column (SEC) Superdex S200 16/60 using an ÄKTA FPLC system (GE Healthcare, Little Chalfont, UK). An additional serine residue remained at the N-terminus of Sx4 and Munc18c proteins after TEV protease treatment (**Fig 1B**). GST-cleaved SNAP23 and VAMP2 were produced and purified as described previously [28]. Briefly proteins were lysed in lysis buffer (25 mM TrisCl, pH7.5, 150 mM NaCl, 0.5% Triton-X 100, 2 mM βME). The lysate was subsequently cleared by centrifugation and incubated with GSH-agarose resin (Thermo-Fisher Scientific, Massachusetts, USA) for 2 hrs. The beads were then washed with wash buffer (25 mM TrisCl, pH7.5, 150 mM NaCl, 2 mM βME) prior to treatment with thrombin to cleave the GST affinity tag. Cleaved protein was further purified by anion exchange chromatography on a MonoQ HR 5/5 column (GE Healthcare, Little Chalfont, UK) for SNAP23 and by cation exchange chromatography on a MonoS HR 5/5 column (GE Healthcare, Little Chalfont, UK) for VAMP2 and VAMP2 E78C.

### Production of HT-Sx4_1-298_-TMD

A single colony was picked from freshly transformed HT-Sx4_1-298_-TMD in Lemo21 (DE3) cells (New England, BioLabs Inc., Ipswich, MA), and used to inoculate 200 mL LB containing antibiotics (50 μg/mL kanamycin). Cultures were incubated overnight at 37°C with shaking and then used to inoculate several l L cultures containing appropriate antibiotics. Cells were incubated at 37°C with shaking (200 rpm) and induced with 1 mM IPTG and 0.25 mM L-rhamnose at an OD_600_ of 0.6. Growth was continued for 18–20 h at 22°C with shaking. Cells were harvested by centrifugation (5000g, 4°C). For protein purification, cells were homogenized in a 1:10 ratio (wet cell pellet mass: buffer) using lysis buffer (25 mM Tris-Cl pH 7.5, 150 mM NaCl, 2 mM βME, 10 mM MgCl_2,_ 1% (v/v) Triton X-100, 1% (w/v) DDM (Glycon Biochemical, Luckenwalde, Germany) 12,500–14,000 units DNase (Roche, Basel, Switzerland), 100 μL of Bacterial Protease Inhibitor (Bio Pioneer, Inc., San Diego, CA). Cells were then lysed by addition of lysozyme (Astral Scientific, Gymea, NSW, Australia) to a final concentration of 200 μg/mL followed by incubation at 4°C for 2 h. Insoluble protein and cell debris were removed by centrifugation using a Beckman Optima™ L-100 XP ultracentrifuge (45Ti rotor, 30, 000g, 40 min, 4°C). The soluble membrane fraction was mixed with pre-equilibrated TALON™ Co^2+^ beads and incubated at 4°C. After extensive washing (25 mM Tris-Cl pH 7.5, 200 mM NaCl, 10% (v/v) glycerol, 0.1% (w/v) β-DDM with 10 mM imidazole, followed by buffer with 25 mM imidazole) the protein was eluted in buffer (25 mM Tris-Cl pH 7.5, 200 mM NaCl, 10% (v/v) glycerol, 0.1% (w/v) β-DDM, 300 mM imidazole) and further purified on Superdex 200 (G10/300) column pre-equilibrated with SEC buffer (25 mM Tris-Cl pH 7.5, 200 mM NaCl, 2 mM βME, 0.03% (w/v) DDM, 10% (v/v) glycerol) on an AKTA FPLC system (GE Healthcare, Little Chalfont, UK). To generate de-tagged protein, HT-Sx4_-1-298_-TMD eluted from the IMAC resin was mixed with TEV protease in a 1:10 ratio (A_280_ ratio of protease: protein) and incubated while being buffer exchanged into 25 mM Tris-Cl pH 7.5, 200 mM NaCl, 2 mM βME, 0.03 (w/v) β-DDM, 10% (v/v) glycerol, 10 mM imidazole overnight at 4°C. A reverse-IMAC purification step was then used to separate the de-tagged membrane protein.

### *In vitro* pull-down binding assays with immobilized Sx4 mutants or immobilized Munc18c

Two pull-down assays were used to assess Sx4 protein binding with Munc18c and SNAREs. In the first set of experiments, all Sx4 proteins (Sx4_1-275_-His_,_ Sx4_1-275_-T4L-His or Sx4_30-275_-T4L-His) (30 μg each, 4.7 μM (Sx4_1-275_-His), 3.0 μM (Sx4_1-275_-T4L-His) or 3.2 μM (Sx4_30-275_-T4L-His) in a volume of 200 μL) were immobilized onto TALON™ Co^2+^ beads via their C-terminal His_6_-tag fusion. Beads bound with Sx4 proteins were mixed with purified de-tagged Munc18c protein (50 μg, 3.7 μM), prepared by TEV protease treatment of HTMunc18c at 4°C for 2 h to assess binary complex formation. For quaternary complexes, beads with immobilized Munc18c/Sx4-His (Sx4_1-275_-His or Sx4_1-275_-T4L-His) complex were incubated with GST-cleaved SNAP23/VAMP2 (40 μg) overnight at 4°C. Binding buffer contained 25 mM Tris-Cl pH 7.5, 150 mM NaCl, 10 mM imidazole, 0.1% (v/v) Triton X-100, 10% (v/v) glycerol, 2 mM βME, 2 mM AEBSF. The beads were washed extensively with binding buffer (excluding 2 mM AEBSF) and the bound proteins analyzed by SDS-PAGE and Coomassie Blue staining. The Sx4-T4L fusion proteins, Sx4_1-275_-T4L-His and Sx4_30-275_-T4L-His were used to assess binary complex formation (Munc18c/Sx4), whereas for quaternary complex (Munc18c/SNARE) formation only the Sx4_1-275_-T4L-His construct was used.

In a second set of experiments, HL-Munc18c (20 μg, 1.4 μM in a volume of 200 μL) was first immobilized on TALON™ Co^2+^ beads. Beads were incubated separately with de-tagged Sx4_1-275_, Sx4_1-275_-T4L or Sx4_1-298_-TMD (50 μg, 21.7 μM) for 4 h in the binding buffer described above and washed with binding buffer. For quaternary complex formation, the beads (containing HL-Munc18c/Sx4 binary complexes or Sx4 proteins without HL-Munc18c for the control) were mixed with purified SNAP23 (100 μg, 21.7 μM)) and VAMP2 (80 μg, 31.7 μM) proteins and incubated overnight at 4°C using each of the three de-tagged Sx4 proteins (Sx4_1-275_, Sx4_1-275_-T4L and Sx4_1-298_-TMD) in binding buffer. Unbound and non-specific proteins were removed by washing with binding buffer and the level of binding was analyzed by SDS-PAGE. Each binding assay was repeated at least three times to confirm the results. Protein concentrations were measured using the Bio-Rad protein assay with bovine serum albumin used as a standard.

Nonspecific binding of detagged proteins was assessed by incubating the individual proteins overnight either with T4-Lysozyme-His (20 μg) immobilized onto beads (**S3 Fig**) or with beads alone (**Fig 4**(for Munc18c)) and **Fig 5**(for Sx41-275, Sx41-298-TMD, or Sx41-275-T4L)).

### Isothermal titration calorimetry (ITC)

ITC experiments were carried out at 298 K using an iTC200 (Malvern Instruments Ltd., Malvern, United Kingdom). The proteins were buffer exchanged in ITC buffer (25 mM HEPES pH 8.0, 200 mM NaCl, 10% (v/v) glycerol and 2 mM β-ME) by gel filtration prior to ITC experiments. Sx4_1-275_-T4L-His or Sx4_30-275_-T4L-His at a concentration of 200–250 μM was titrated into 20–40 μM of HMunc18c in the cell. Injection volumes of 2.5 μL were used for all titrations. The heat released was measured and integrated using the Microcal Origin 7.0 program with a single site binding model to calculate the equilibrium association constant *K*_*a*_ *(= 1/K*_d_*)*, enthalpy of binding (Δ*H*) and the stoichiometry (*n)*. The Gibbs free energy *(*Δ*G)* was calculated using the equation:

$$\Delta G=-RTln(K_{a})$$

Binding entropy (Δ*S*) was calculated by

ΔG=ΔH−TΔS.

To determine the average standard error of the mean (SEM) values of binding affinities, four experiments were performed for each set of samples.

### Bio-layer interferometry

The binding kinetics of Sx4_1-275_ or Sx4_1-275_-T4L for their interaction with Munc18c was determined by bio-layer interferometry using a BLItz^®^ system (ForteBio, Menlo Park, CA). In the first series of experiments C-terminally His-tagged Sx4 constructs (200–400 nM) were loaded onto Ni-NTA biosensors until a binding height of ~ 1 nm was reached. Sx4 immobilized sensors were dipped into a dilution series of de-tagged Munc18c at concentrations of 25, 50, 100, 200 and 400 nM for Sx4_1-275_-His and 10, 25, 50, 100, 200 and 400 nM for Sx4_1-275_ -T4L-His immobilized biosensors. The association reaction was allowed to proceed for 60–100 s and the dissociation reaction for 120–180 s. Experiments were conducted with a shaking speed of 2200 rpm in 25 mM Tris-Cl pH 7.5, 150 mM NaCl, 10 mM imidazole, 0.1% (v/v) TX-100, 10% (v/v) glycerol and 2 mM βME buffer. We were unable to assess the association of Munc18c with Sx4_1-298_-TMD using biolayer interferometry because of poor yield and purity of C-terminally His-tagged full-length Sx4 protein.

In a second series of experiments HL-Munc18c was immobilized onto Ni-NTA biosensors until a binding height of ~ 1 nm was achieved. The biosensors were dipped into a concentration series of 25, 50, 100, 200 and 400 nM for detagged Sx4_1-275_ and 25, 50, 100, 200 and 400 nM for detagged Sx4_1-275_ -T4L. The association reaction was for 100 s and dissociation was followed for 120 s. Experiments were conducted three times using at least two replicates from independently prepared samples and the data were fitted to an interaction with 1:1 stoichiometry using BLItz^®^ Pro Software.

### Labeling proteins for fluorescence experiments

Cysteine variants were prepared using QuikChange Mutagenesis (Stratagene, San Diego, CA). These were (i) Sx4_1-275_-His and Sx4_1-275_-T4L (native C141 rather than C141S), to study Munc18c/Sx4 kinetics and (ii) GST cleaved VAMP2 E78C to study SNARE ternary complex formation. Site-specific labeling of these two proteins was performed using the thiol reactive Alexa488 C_5_ Maleimide dye according to the manufacturer’s instructions (Alexa Fluor® 488, Life Technologies, Carlsbad CA). Briefly, purified protein (200 μM) was mixed with dye to give a final dye concentration of 1 mM (in DMSO, final concentration 10% v/v) and incubated for 2–3 h in the dark at room temperature. Unbound dye was removed by sequentially passing the protein-dye mix through two equilibrated desalting PD10 columns (GE Healthcare, Little Chalfont, UK). The protein was collected and concentrated to the desired concentration. The absorbance of labeled protein at 280 nm (*A*_280_) and 494 nm (*A*_494_) was measured using a 1 cm cuvette in a nanodrop, and the degree of labeling was then calculated as recommended by the supplier:

$$\mathrm{degree}\;\mathrm{of}\;\mathrm{protein}\;\mathrm{labeling}=\frac{A_{494}\cdot \varepsilon_{\mathrm{protein}}}{\varepsilon_{\mathrm{Alexa}-488}\left(A_{280}-0.11\times A_{494}\right)}\times 100\%$$

where the approximate molar extinction coefficient of Alexa 488 (*ε*_Alexa-488_) at 494 nm is 71000 M^-1^cm^-1^. The molar extinction coefficient of the protein (*ε*_protein_) was calculated from each protein sequence using the Expasy Protparam server [47]. In all cases, the degree of labeling was calculated to be greater than 90%.

### Fluorescence anisotropy measurements

Long-time base-fluorescence anisotropy measurements were performed at 25°C in 1 mL quartz cuvettes (Hellma, Mullheim, Germany) without stirring on a Fluoromax-4 spectrofluorometer fitted with polarizers of L-geometry and slits set at 4 nm (Jobin Yvon Inc., Edison, NJ). The experiments were carried out in a buffer containing 25 mM HEPES pH 8.0, 200 mM NaCl, 1 mM TCEP and 2% (w/v) glycerol. The Alexa488 fluorophore was excited at 488 nm and emission was observed at 515 nm. Fluorescence anisotropy (*r*) was determined as:

$$<r>=I_{VV}-G\;I_{VH}∕I_{VV}-2GI_{VH}$$

where *I* represents the fluorescence intensity, and the first subscript letter indicates the direction of excited light and second subscript shows the emitted light. The intensities of vertically (*V*) and horizontally (*H*) polarized emission light after excitation were measured. The “G-factor” *G* was defined as:

$$G=I_{VH}∕I_{HH}$$

Primary data analysis was performed with the programs Graffit 5.0 (Erithacus software) and Fluorescence implementation (Jobin Yvon Inc., Edison, NJ) of the Origin7.0 software (Origin lab Corporation, Northampton, MA). Experiments investigating the kinetics of the interaction between Sx4_1-275_-His or Sx4_1-275_-T4L-His and HMunc18c were performed once at each concentration of HMunc18c. The experiments investigating the formation of the SNARE complex used Alexa488-labeled VAMP2 (GST-cleaved), GST cleaved SNAP23, and the same Sx4 and Munc18c proteins, and were each performed at least three times. All spectra were corrected for background fluorescence from buffer.

To confirm the specificity of binding and complex formation, all proteins (Sx4_1-275_-His, Sx4_1-275_ -T4L-His, HMunc18c, GST-cleaved SNAP23) were tested individually for their ability to bind Alexa488-labeled VAMP2 (GST cleaved). The change in anisotropy was monitored and showed that none of these proteins interacted on their own with VAMP2-Alexa488 under the given experimental conditions. We were unable to assess the association of HMunc18c with the full length Sx4_1-298_-TMD-His using fluorescence anisotropy because the presence of detergent prevented a stable baseline under the same conditions.

The global fit for the anisotropy data was calculated using the Dynafit 4.0 program. During the fitting procedure (1:1 complex), the concentration of proteins was allowed to vary and then checked against the anticipated value. The script used in global calculations of kinetic parameters for the interaction of Munc18c with Sx4 or Sx4-T4L is provided as supporting information (**S1 Text**).

## Supporting information

S1 Table Thermodynamic parameters for the HMunc18c:Sx4_1-275_-T4L-His and HMunc18c:Sx4_30-275_-T4L-His interactions determined using isothermal titration calorimetry. Values are shown as mean ± SEM from four independent experiments. NB indicates no binding was detected.
(PDF)

S1 Fig ITC data for the interaction between HMunc18c and Sx4_1-275_-T4L-His and HMunc18c and Sx4_30-275_-T4L-His. The upper panel depicts the raw ITC data while the lower panel is the integrated normalized data. Experiments were conducted at 298 K where Sx4_1-275_-T4L-His or Sx4_30-275_-T4L-His (200–250 μM) was titrated into HMunc18c (20–40 μM) in the cell with an injection volume of 2.5 μL.
(EPS)

S2 Fig **Bio-layer interferometry data for interaction between Sx4 constructs and Munc18c (A)** immobilized Sx4_1-275_-His with Munc18c in solution, **(B)** immobilized Sx4_1-275_-T4L-His with Munc18c in solution, **(C)** HLMunc18c immobilized with Sx4_1-275_ in solution and **(D) HL**Munc18c immobilized with Sx4_1-275_-T4L in solution. The black line denotes the fitting for a 1:1 binding model.
(EPS)

S3 Fig Immobilized T4L does not interact with (detagged) Sx4, Munc18c, SNAP23 or VAMP2. Coomassie Blue stained SDS-PAGE gels. Lanes labeled INPUTS show the protein samples used in the experiments and the T4L-His protein immobilized on resin. Lanes labeled Controls show the interaction between T4L and the detagged SNARE and Munc18c (M18c) proteins.
(EPS)

S1 Text Script for global calculation of kinetic parameters by fluorescence anisotropy. (PDF)
