# Supplementary material for: The nature of the Syntaxin4 C-terminus affects Munc18c-supported SNARE assembly
Source: PLoS One. 2017 Aug 25;12(8):e0183366. doi: 10.1371/journal.pone.0183366 (PMC5571939; doi:10.1371/journal.pone.0183366)
Supplement: S1 Table — Values are shown as mean ± SEM from four independent experiments. NB indicates no binding was detected. (PDF) [file pone.0183366.s001.pdf]

**S1 Table. Thermodynamic parameters for the HMunc18c:Sx4$_{1\text{-}275}$-T4L-His and HMunc18c:Sx4$_{30\text{-}275}$-T4L-His interactions determined using isothermal titration calorimetry.** Values are shown as mean ± SEM from four independent experiments. NB indicates no binding was detected.

| **HMunc18c: Sx4$_{1\text{-}275}$-T4L-His and HMunc18c: Sx4$_{30\text{-}275}$-T4L-His thermodynamic parameters** | | | | | | |
|---|---|---|---|---|---|---|
| **In cell** | **Titrant** | **ΔH (kcal/mol)** | **TΔS (kcal/mol)** | **ΔG (kcal/mol)** | **$K_d$ (nM)** | **N** |
| HMunc18c | Sx4$_{1\text{-}275}$-T4L-His | -10.4 ± 0.9 | 5.2 ± 1.3 | -9.5 ± 0.3 | 100 ± 40 | 1.0 |
| HMunc18c | Sx4$_{30\text{-}275}$-T4L-His | NB | NB | NB | NB | NB |
