# Supplementary material for: The nature of the Syntaxin4 C-terminus affects Munc18c-supported SNARE assembly
Source: PLoS One. 2017 Aug 25;12(8):e0183366. doi: 10.1371/journal.pone.0183366 (PMC5571939; doi:10.1371/journal.pone.0183366)
Supplement: S1 Text — (PDF) [file pone.0183366.s005.pdf]

S1 Text: Script for Syntaxin4:Munc18c interaction

[task]
data = progress task = fit

[components] A,B; 'Munc', 'Sx'

[mechanism] A+B<===>A.B:kon koff

[constants] kon = 1.1e3? koff = 0.0005?

[responses] B=4.76034e+006 A.B=5.55039e+006

[concentrations]
;B=50e-9; all in [data] section

[data]
delay 15
error constant 0.05
directory C:\Users\BioAdmin\Documents\my\141105\data extension txt

file 100 file 200c file 300c file 400c file 1000 file 1500c file 2000c file 2500

[settings]

{Output} StartDefaultBrowser = n

| conc B=50e-9? | conc A=100e-9?, | offset= 0.238?

| conc B=50e-9? | conc A=200e-9?, | conc B=50e-9? | conc A=300e-9?, | conc B=50e-9? | conc A=400e-9?,

|offset= 0.237? |offset= 0.239? |offset= 0.239?

| conc B=50e-9? | conc A=1000e-9?, | conc B=50e-9? | conc A=1500e-9?, | conc B=50e-9? | conc A=2000e-9?,

| conc B=50e-9? | conc A=2500e-9?,

|offset= 0.239? |offset= 0.233? |offset= 0.239?

|offset= 0.240?

[output]
directory C:\Users\BioAdmin\Documents\my\141105\output-sx4-publication

[end]

script for Syntaxin4-T4L:Munc18c interaction

[task]
data = progress

task = fit

[components] A,B; 'Munc', 'Sx'

[mechanism] A+B<===>A.B:kon koff

[constants] kon = 600? koff = 4.6e-5?

[responses] B=4.94034e+006 A.B=5.71339e+006

[concentrations]
;B=50e-9; all in [data] section

[data]
error constant 0.05
directory C:\Users\BioAdmin\Documents\my\141203\data extension txt

file 100 file 200 file 400 file 600 file 800 file 1000 file 1500

[settings]

{Output} StartDefaultBrowser = n

| conc B=50e-9? | conc A=100e-9?, | offset= 0.243?

- | conc B=50e-9? | conc A=200e-9?,
- | conc B=50e-9? | conc A=400e-9,
- | conc B=50e-9? | conc A=600e-9?,

|offset= 0.241? |offset= 0.240?

|offset= 0.244? | conc B=50e-9? | conc A=800e-9?, |offset= 0.244?

| conc B=50e-9? | conc A=1000e-9?, | conc B=50e-9? | conc A=1500e-9?,

|offset= 0.244? |offset= 0.244?

[output]
directory C:\Users\BioAdmin\Documents\my\141203\output-sx4t4-for-paper [end]
